# Comprehensive Human Papillomavirus Genotyping in Cervical Squamous Cell Carcinomas and Its Relevance to Cervical Cancer Prevention in Malawian Women

**DOI:** 10.1200/JGO.2015.001909

**Published:** 2016-08-10

**Authors:** Brooke E. Howitt, Michael Herfs, Tamiwe Tomoka, Steve Kamiza, Tarik Gheit, Massimo Tommasino, Philippe Delvenne, Christopher P. Crum, Danny Milner

**Affiliations:** **Brooke E. Howitt**, **Christopher P. Crum**, and **Danny Milner**, Brigham and Women’s Hospital, Boston, MA; **Michael Herfs** and **Philippe Delvenne**, University of Liege, Liege, Belgium; **Tamiwe Tomoka**, **Steve Kamiza**, and **Danny Milner**, Malawi College of Medicine, Blantyre, Malawi; and **Tarik Gheit** and **Massimo Tommasino**, International Agency for Research on Cancer, Lyon, France.

## Abstract

**Purpose:**

Cervical squamous cell carcinoma (SCC) continues to be a significant cause of cancer morbidity and is the third leading cause of cancer-related death in women worldwide. In sub-Saharan Africa, cervical cancer is not only the most common female cancer but also the leading cause of cancer-related deaths in women. Malawi, in particular, has the highest burden of cervical cancer. With the increasing use of human papillomavirus (HPV) vaccination, documenting the prevalent HPV types in those high-risk populations is necessary to both manage expectations of HPV vaccination and guide future vaccine development.

**Materials and Methods:**

In this study, we performed HPV typing on 474 cervical SCC samples and analyzed the potential impact of HPV vaccination in this population.

**Results:**

Ninety-seven percent of tumors were positive for at least one HPV type, and 54% harbored more than one HPV type. HPV 16 was the most common type (82%), followed by HPV 18 (34%), HPV 35 (24%), and HPV 31 (12%). A vaccine against HPV 16 and 18 would ideally prevent 53% of cervical SCC, and the nonavalent HPV vaccine (covering HPV 16, 18, 31, 33, 45, 52, and 58) would prevent 71% of cervical SCC in Malawi (assuming 100% vaccine efficacy). The main reason for a lack of coverage was high prevalence of HPV 35, which was also present as a single infection in a small subset of patients.

**Conclusion:**

Although any HPV vaccination in this population would likely prevent a significant proportion of cervical cancer, the nonavalent vaccine would provide better coverage. Furthermore, investigation of the role of HPV 35 in this population, including possible cross-protection with other HPV types, should be pursued.

## INTRODUCTION

Cervical squamous cell carcinoma (SCC) continues to be a significant cause of cancer morbidity and is the third leading cause of cancer-related death in women worldwide^1,2^; the majority of this cancer burden occurs in resource-poor settings, including sub-Saharan Africa (SSA), where cervical cancer is not only the most common female cancer but also the leading cause of cancer-related deaths in women.^3,4^ Within SSA, Malawi has the highest cervical cancer rate, with an incidence of 75.9 per 100,000 women,^1,2^ and cervical cancer comprises 45.4% of all female cancers.^5^ More than 95% of cervical cancer is associated with oncogenic human papillomavirus (HPV) infection, of which there are at least 13 different high-risk HPV (hrHPV) types.^6-14^ Despite the high incidence of cervical SCC in Malawi, the specific HPV types causing tumors in this population have not been previously evaluated on cervical cancer pathology specimens. With the advent of prophylactic vaccination for specific HPVs (Cervarix [recombinant bivalent human papillomavirus vaccine]; GlaxoSmithKline Biologicals, Rixensart, Belgium), Gardasil (recombinant quadrivalent human papillomavirus; Merck, Whitehouse Station, NJ), and Gardasil-9 (recombinant 9-valent human papillomavirus vaccine; Merck, Whitehouse Station, NJ), the HPV type-specific distribution in target populations becomes particularly germane to both expectations from large-scale vaccination and anticipated clinical burden. Bivalent human papillomavirus vaccine and recombinant human papillomavirus cover HPV 16 and 18 (recombinant human papillomavirus also covers the low-risk HPV 6 and 11 that cause genital warts), and 9-valent human papillomavirus vaccine covers the low-risk HPV 6 and 11, in addition to seven high-risk HPVs (16, 18, 31, 33, 45, 52, and 58).^15^ Although HPV 16 and 18 are the most common genotypes worldwide, including in SSA,^16,17^ studies have suggested that some HPV types that are relatively uncommon in Western countries (e.g., HPV 35, 45, 52, 56, and 58) might have a much higher prevalence in resource-poor settings, especially SSA.^3,9,18-24^ Furthermore, an immunocompromised state, most notably, HIV infection, may also affect the number and distribution of HPV genotypes in a given population^3,21,22,24-31^ and confound the estimations of influence exerted by each HPV type in a mixed infection.

The goals of this study were to determine the number and genotypes of HPV present in cervical SCC specimens from Malawian women, and determine the relative percentage of the cervical cancer population predicted to benefit from HPV 16 and 18 vaccination versus the more recent nonavalent vaccine (9-valent human papillomavirus vaccine).

## MATERIALS AND METHODS

### Patient Selection

This study was performed with approval from the institutional review boards at the Malawi College of Medicine and Brigham and Women's Hospital. Patients were selected by reviewing pathology reports from the Malawi College of Medicine in Blantyre, Malawi, to identify invasive SCCs of the uterine cervix. Approximately 500 patients with cervical SCC diagnosed from 2009 to 2012 with available tumor blocks were retrieved from Blantyre. When the age and HIV status of the patient was provided in the pathology report, this information was recorded. Stage, clinical outcome, and survival data were not available.

### Sample Histology Review

A hematoxylin and eosin–stained slide was made from each retrieved block, and all slides were reviewed (B.E.H.) to confirm the diagnosis of invasive SCC and adequacy of the block for DNA extraction. Tumors were evaluated for grade (well, moderate, or poorly differentiated) and whether they demonstrated keratinizing and/or basaloid features.^32^

### DNA Extraction

Genomic DNA was extracted from 474 samples of formalin-fixed, paraffin-embedded tumor tissue using the NucleoSpin DNA FFPE XS kit (Macherey-Nagel, Düren, Germany) according to the manufacturer’s instructions.

### HPV Genotyping

All samples were interrogated for 21 HPV genotypes (6, 11, 16, 18, 26, 31, 33, 35, 39, 45, 51, 52, 53, 56, 58, 59, 66, 68^a,b^, 70, 73, and 82) using the type-specific E7 polymerase chain reaction bead-based multiplex assay (TS-E7-MPG, International Agency for Research on Cancer, Lyon, France).^33,34^ This ultrasensitive HPV genotyping assay, allowing detection of fewer than 10 copies of the viral genome, was previously validated by a WHO LabNet proficiency panel.^33,35^ Two beta-globin primers were included to control for DNA quality. The 21 HPV genotypes included all of those previously reported in cervical cancers at a frequency of 1% or greater.^36^

## RESULTS

### Patient Population and Histologic Features

Patient age was reported for 395 patients (80%), with a median age of 45 years (range, 24 to 96 years; 25th percentile, 37 years; 75th percentile, 56 years). Of the 15 patients for whom HIV status was documented on the pathology requisition form, all were reportedly positive for HIV; however, insufficient data were available to understand the complete role/relationship of HIV and cervical cancer in this population. On the basis of the prevalence of HIV in Malawi,^5,37-39^ we assume a high rate of HIV seropositivity in this cohort but were not able to confirm it. The number of patients per year (103 from 2009; 110 from 2010; 130 from 2012; and 131 from 2011) and the median age of patients per year (2009 median, 43 years [n = 89]; 2010 median, 44 years [n = 99]; 2011 median, 45 years [n = 103]; and 2012 median, 45 years [n = 104]) were similar.

SCC was confirmed in 474 patients. Eighteen samples (3.8%) were well differentiated, 250 (53.2%) were moderately differentiated, and 202 (43%) were poorly differentiated. In four samples, the tissue preservation was poor, precluding this evaluation. There was no significant association between age and tumor grade.

### HPV Genotyping

**Fig 1:**
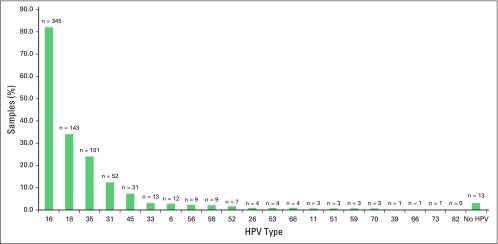
Prevalence and types of human papillomavirus (HPV) across all cervical squamous cell carcinomas. The HPV types identified across all samples, presented as percentage (green bars; vertical axis) of total samples and absolute number (displayed above green bar) against HPV type (horizontal axis), regardless of single or multiple infections.

**Fig 2:**
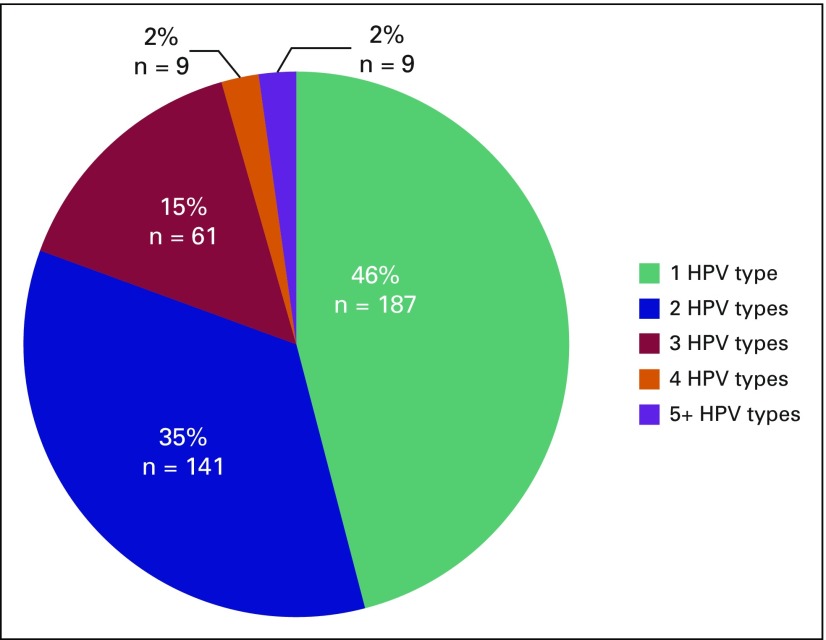
Summary of prevalence of human papillomavirus (HPV) single and multiple infections in 407 high-risk HPV cervical squamous cell carcinomas. More than half of the patients demonstrated more than one HPV type, whereas 46% had one HPV type (green). In patients with more than one HPV type identified, the majority harbored two HPV types (blue); however a significant subset (red, orange, purple) harbored three or more HPV types.

The amplification of the beta-globin gene showed that good-quality DNA could be obtained for 420 (89%) of the 474 paraffin-embedded tissue samples. The 54 samples (11%) negative for the beta-globin test were thus excluded from the remaining analyses. Of the 420 samples, 407 (97%) were positive for at least one hrHPV type (Fig 1). Either as a single infection or as part of multiple infections, HPV 16 was the most common genotype detected, present in 82% of samples followed by HPV 18 (34%), HPV 35 (24%), and HPV 31 (12%; Fig 1). Although 46% of samples had only one HPV type, the majority (54%) of tumors harbored more than one HPV type, and 19% had three or more HPV types (Fig 2; Table 1). Low-risk HPV types 6 and 11 were present in 2.8% and 0.7% of samples, respectively (Fig 1), but were always accompanied by at least one hrHPV type. All HPV types interrogated were detected in at least one sample, with the exception of HPV 82. The known HIV-positive samples were associated with one to four HPV types (mean and median, 2), which was not different from the undocumented samples. Of the 14 HIV-positive samples, 12 harbored HPV 16, eight of which also harbored another hrHPV type. HPV 31 and HPV 18 were also present as single infections (one sample each) in the documented HIV-positive group.

**Table 1:**
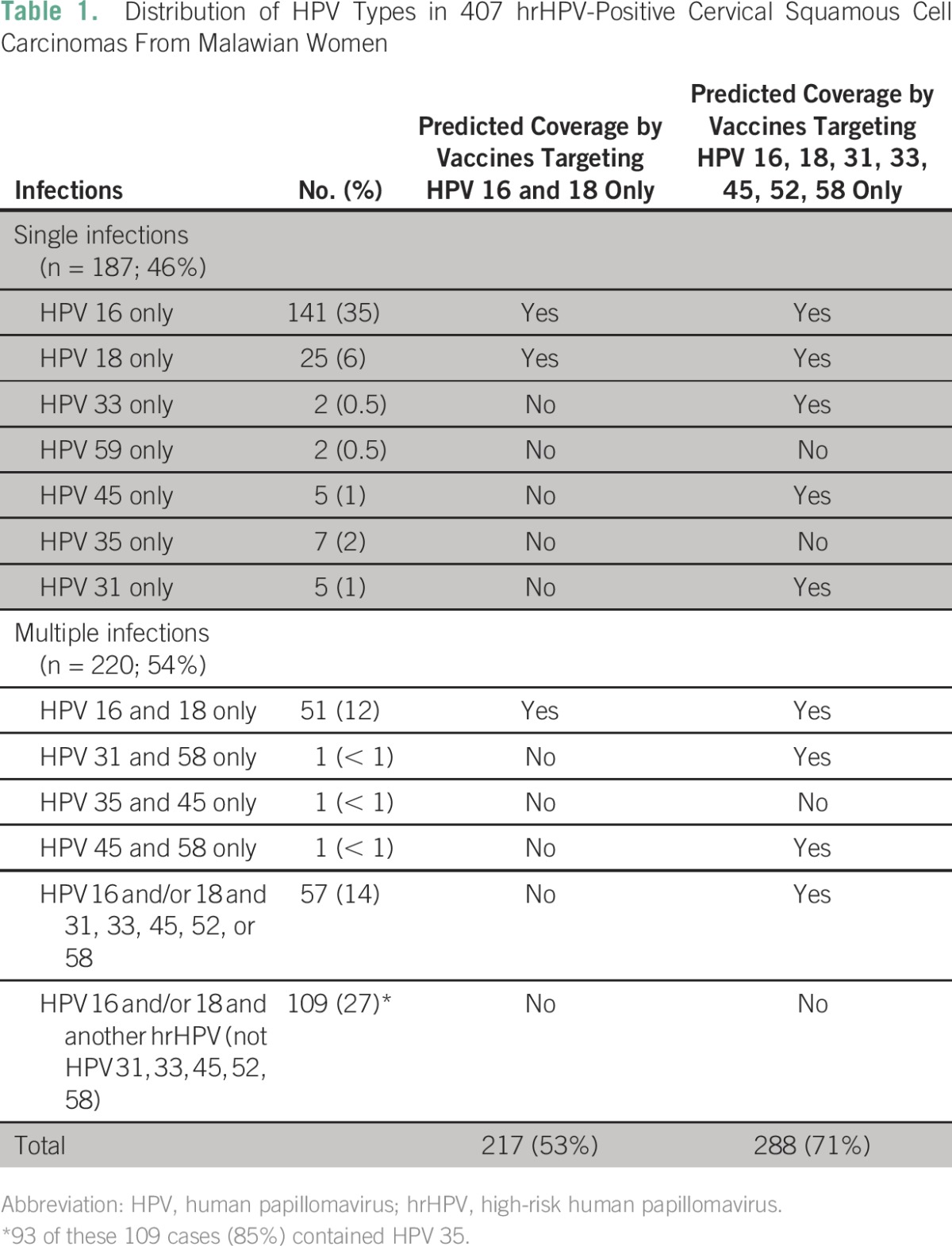
Distribution of HPV Types in 407 hrHPV-Positive Cervical Squamous Cell Carcinomas From Malawian Women

### Prediction of HPV Vaccine Coverage

In considering relative efficacy of the various available vaccines, for this limited analysis, we assumed 100% protection for the hrHPV types included in the available vaccines and no cross-protection between different HPV types. Of the HPV-positive samples (n = 407), 53% had only HPV 16 and/or HPV 18 genotypes detected, suggesting that these cancers would be preventable with vaccination specifically for HPV 16 and 18 only. However, 47% of samples in this cohort harbored other high-risk HPV types in addition to or instead of HPV 16 and 18 (Table 1), suggesting that a significant proportion of this population would benefit from broader vaccine coverage. In considering the 9-valent human papillomavirus vaccine, which covers HPV 6, 11, 16, 18, 31, 33, 45, 52, and 58, 71% of patients harbored HPV genotypes entirely covered by this vaccine. Of the remaining 29% who would be uncovered, the majority (83%) harbored HPV 35, which is not included in any known HPV vaccine. The addition of HPV 35 coverage to the types covered in 9-valent human papillomavirus vaccine would provide a total predicted coverage of 95%.

## DISCUSSION

There is an abundance of literature on HPV genotyping, which has revealed striking geographic differences in HPV type-specific prevalence among continents, as well as among countries within the same continent. Although infection with only one hrHPV genotype is common in the United States, in some SSA countries, nearly 70% of cervical cancers contain multiple hrHPV genotypes.^16,40,41^ In Malawi, cervical cancer is the most common malignancy in women, representing 42% to 45% of all female cancers; survival from cervical cancer is dismal, with a median survival of 9 months and 1-year, 2-year, 3-year, and ≥ 4-year survival of 31.0%, 10.3%, 5.2%, and 2.9%, respectively.^5,37^ If given the opportunity, there was unanimous agreement among Malawian women on choosing to vaccinate their children against HPV, suggesting that HPV vaccination would be well received in this population.^42^

HPV genotypes in cervical cancer have not been previously evaluated in Malawi, and with the possible implementation of HPV vaccination, we thought it would be important to document the baseline HPV genotype prevalence in cervical cancers in this population, particularly with respect to the predicted benefit that a vaccine covering just the high-risk HPV 16 and HPV 18 might confer compared with the newer and broader 9-valent human papillomavirus nonavalent vaccine that covers seven hrHPVs, including HPV 16 and HPV 18. Our study has shown that although HPV 16 and HPV 18 are the most common types in Malawi cervical SCC, multiple coinfections are common, and only 53% of the population could be protected by the administration of recombinant human papillomavirus or bivalent human papillomavirus (assuming no cross-protection, which is discussed in further detail later). Many researchers who have undertaken similar studies or meta-analyses evaluating the potential efficacy of the available vaccines did not take into account HPV 35.^43^ Data on the rates of HPV 35 positivity in cervical cancers and precursors are limited; however, in most reported countries, the rate is less than 6%.^3,23,44-48^ Some have proposed that HPV 35 may have a lesser oncogenic potential compared with HPV 16 and 18.^49^ Interestingly, our results align closely with a recent study evaluating HPV types present in HIV-positive Malawian women (not limited to cancer). Although the number of cancers in that study was small (n = 42 with abnormal acetowhite visual inspection, including 29 women with presumed invasive cancer), the researchers found that 33% of clinically abnormal cervices harbored HPV 35, but this HPV genotype was detected in only 6% of visually normal cervices,^50^ suggesting that HPV 35 is indeed carcinogenic but may be more important in the setting of HIV positivity. Indeed, 40% of HIV-positive women from Burkina Faso and Botswana tested positive for HPV 35.^23,24^ In Table 1, 27% of patients had HPV 16 and/or 18, in addition to another non-HPV 31, 33, 45, 52 or 58 type (which most frequently was HPV 35). However, we recognize that most of these cancers may be primarily driven by HPV 16 and/or 18. Although no strong conclusion can be made regarding HPV 35 and our study population, it is intriguing that we have identified a high prevalence of this otherwise uncommon hrHPV, and future studies regarding the oncogenicity of HPV 35 are warranted.

In this study, we were limited by our lack of knowledge of HIV status in the majority of patients; however, on the basis of the literature, at least 50% of Malawian women with cervical cancer may be estimated to be HIV positive.^5,37-39^ It would have been interesting to know whether those cancers in HIV-positive women were more likely to be associated with multiple hrHPV types, as has been demonstrated in studies in Zambia, Botswana, and South Africa, which showed a median of two to four hrHPVs present in HIV-positive women.^26-28,51^ Similarly, we were unable to assess whether specific HPV genotypes, particularly HPV 35, were associated with HIV infection. Additionally, this study was unable to assess the prevalence of HPV genotypes in women with normal cervical examinations or only precursor lesions; therefore, we were unable to determine a weighted attribution of individual HPV types to the development of invasive cancer.

Some data exist regarding potential cross-protection in HPV 16 and 18 vaccines with non-16 and 18 HPV types, with the strongest evidence for cross-protection for HPV 31 from both bivalent human papillomavirus vaccine and recombinant human papillomavirus^52^; however, this effect may wane with time, because the strongest cross-protective effect was demonstrated in trials with the shortest follow-up time,^53^ and it was relatively less efficacious in those studies with longer follow-up periods.^54^ Although there is limited (but conflicting) evidence for some cross-protection against HPV 33, 45, 52, and 58 from the HPV 16 and 18 vaccines, notably, there has been no reported efficacy against HPV 35,^55^ although serum antibodies to HPV 35 have been documented after HPV vaccination.^56^ The precise impact of HPV 35 is unclear because it is present as a single infection in only a fraction of patients. Importantly, in the context of HIV positivity, studies have shown that immunogenicity and cross-protective serologic responses have been documented against HPV 31, 33, and 45 in HIV-positive patients,^57-60^ suggesting that HPV vaccination should be effective in this subpopulation.

In considering a more widespread use of HPV vaccination, particularly in resource-poor countries, cost effectiveness is important.^61^ Recently, groups have analyzed two-dose versus three-dose schedules for administration of bivalent human papillomavirus vaccine and recombinant human papillomavirus, and found that a two-dose schedule is more cost effective, unless the cost of the third dose is significantly decreased, and have even raised the possibility of exploring the efficacy of one-dose schedules. However, this analysis has not yet been performed on the new 9-valent human papillomavirus nonavalent vaccine.^62,63^ Another potential method in cost-effective prevention of cervical cancer, particularly in resource-poor countries, includes the recently reintroduced concept of prophylactic ablation of the cervical squamocolumnar junction.^64-66^ The squamocolumnar junctions are thought to be the cells initially infected by HPV and thus give rise to clinically significant precursor lesions and carcinoma; by ablating this region, the potential to develop lesions is drastically reduced, regardless of the HPV type present.^67-69^ However, the feasibility, safety, and efficacy of this approach have yet to be proven.

In conclusion, this study is the first large study characterizing HPV genotypes present in invasive SCC of the cervix in Malawian women. Our study has shown that although any HPV vaccination will likely have a significant effect on the future incidence of invasive cervical cancer, if given the option, broader vaccine coverage might be more beneficial in this population. Furthermore, the high prevalence of HPV 35 suggests that a significant subset of cancers may not be prevented with any currently available HPV vaccine. This study highlights the importance of studying cross-protection against HPV 35 with the clinically available HPV vaccines. If additional future multivalent vaccines are to be designed, a careful evaluation of HPV genotypes present in invasive cancer in those countries harboring the highest burden of disease, and thus having the most to gain from a broader HPV vaccine, should be considered.
